# Effect of optimized new Shengmai powder on exercise tolerance in rats with heart failure by regulating the ubiquitin-proteasome signaling pathway

**DOI:** 10.3389/fcvm.2023.1168341

**Published:** 2023-05-23

**Authors:** Shuai Wang, Lin Wang, Shaoke Gu, Yixiao Han, Linfeng Li, Zhuangzhuang Jia, Ning Gao, Yu Liu, Shanshan Lin, Yazhu Hou, Xianliang Wang, Jingyuan Mao

**Affiliations:** ^1^ First Teaching Hospital of Tianjin University of Traditional Chinese Medicine, National Clinical Research Center for Chinese Medicine Acupuncture and Moxibustion, Tianjin, China; ^2^ Graduate School, Tianjin University of Traditional Chinese Medicine, Tianjin, China; ^3^ Department of Geratology, Shijiazhuang Hospital of Traditional Chinese Medicine, He Bei, China; ^4^ Department of Cardiology, ShenZhen Traditional Chinese Medicine Hospital, Shen Zhen, China

**Keywords:** ubiquitin-proteasome, heart failure, exercise tolerance, skeletal muscle, optimized new Shengmai powder

## Abstract

**Introduction:**

Decreased exercise tolerance is a common symptom in patients with heart failure, which is closely related to protein degradation and apoptosis regulated by the ubiquitin-proteasome signaling (UPS) pathway. In this study, the effect of Chinese medicine, optimized new Shengmai powder, on exercise tolerance in rats with heart failure was investigated via the UPS pathway.

**Methods:**

The heart failure model was prepared by ligating the left anterior descending branch of the coronary artery in rats, in which the sham-operated group was only threaded and not ligated. Rats (left ventricular ejection fraction ≤ 45%) were randomly divided into the following groups: model group, YHXSMS group, Benazepril group, and proteasome inhibitor Oprozomib group, and they were administered the corresponding drugs by gavage for 4 weeks. The cardiac function of rats was evaluated by performing an echocardiography examination and a hemodynamic test and the exercise tolerance was done by conducting an exhaustive swimming test. The mechanism was revealed by TUNEL detection, immunohistochemistry, immunofluorescence analysis, Western blot, and quantitative real-time PCR.

**Results:**

The study showed that there was a decrease in cardiac function and exercise tolerance of rats in the model group and also destruction of cardiac and skeletal muscle fibers, a proliferation of collagen tissue, and an increment of apoptosis. Our study suggested that optimized new Shengmai powder could exert antiapoptotic effects on myocardial and skeletal muscle cells and improve myocardial contractility and exercise tolerance by inhibiting the overactivation of the UPS pathway, downregulating MAFbx, and Murf-1 overexpression, inhibiting the activation of the JNK signaling pathway, upregulating bcl-2 expression, and decreasing bax and caspase-3 levels.

**Conclusions:**

The study showed that the optimized new Shengmai powder could improve cardiac function and exercise tolerance in rats with heart failure through the UPS pathway.

## 1. Introduction

Heart failure is the end-stage of various heart diseases, and the number of patients with heart failure is increasing in the context of aging, with a 5-year survival rate comparable to malignant tumors (1). As one of the main symptoms of heart failure, decreased exercise tolerance seriously affects the quality of life and prognosis of patients (2, 3), which has become one of the focal areas of concern in heart failure treatment. Although many classical medicines for heart failure also influence exercise capacity, the improvement is limited (4, 5). In addition, although rehabilitation training can restore the muscle strength of patients to some extent and partially improve the prognosis (6, 7), not every patient is able to tolerate and persist with it. Currently, exercise tolerance and quality of life in patients with heart failure remain poor (8).

Many factors contribute to decreased exercise tolerance in patients with heart failure, including impaired cardiac and pulmonary reserves, which fail to deliver adequate amounts of oxygen to muscle tissues, as well as the abnormal function and metabolism of the skeletal muscle (such as the transition from the skeletal muscle fiber type I to type II) (9), myocyte apoptosis, and mitochondrial dysfunction (10, 11). However, the mechanisms that regulate abnormal skeletal muscle function and abnormal metabolism in heart failure remain unclear. It is worth noting that improving cardiac function by modifying ventricular remodeling alone is not equivalent to the corresponding improvement in skeletal muscle function, suggesting the presence of non-cardiogenic mechanisms leading to decreased exercise tolerance in patients with heart failure (12, 13). In addition, a wasting mechanism, called “skeletal muscle atrophy” caused by reduced exercise in heart failure, has been proposed, but exercise training only partially influences the changes occurring in the skeletal muscle (14, 15). At present, the mechanisms underlying the decline in skeletal muscle function in heart failure are still being explored.

There are specifically expressed ubiquitin ligases in both cardiac and skeletal muscles (16) that can regulate several vital activities such as protein degradation and apoptosis through the ubiquitin-proteasome signaling (UPS) pathway (17, 18) and participate in pathological processes such as ventricular remodeling (19–22) and skeletal muscle atrophy (23–25). It is suggested that the UPS may synchronize the regulation of cardiac and skeletal myopathy and play an important role in the decrease of exercise tolerance in patients with heart failure, which deserves further investigation.

Traditional Chinese medicine has advantages in the treatment of heart failure (26, 27) and for improvement in exercise tolerance (28). Optimized new Shengmai powder is a common prescription for treating heart failure in the First Teaching Hospital of Tianjin University of Traditional Chinese Medicine. It mainly consists of *Astragalus membranaceus*, *Salvia miltiorrhiza*, *Semen lepidii*, *Radix codonopsis pilosulae*, *Acanthopanax senticosus*, and others, which has the alleged effect of benefiting Qi, invigorating blood circulation, and promoting diuresis. The previous major science and technology project for “Significant New Drugs Creation” (No. 2010ZX09102-202) showed that the optimized new Shengmai powder could significantly reverse ventricular remodeling, increase cardiac systolic function (29), and improve exercise tolerance (28) in patients with heart failure with few side effects. However, its mechanism is unclear. Therefore, this study investigates the mechanism of the optimized new Shengmai powder in improving exercise tolerance in rats with heart failure by regulating the UPS pathway.

## 2. Materials and methods

### 2.1. Animal

**Figure 1:**
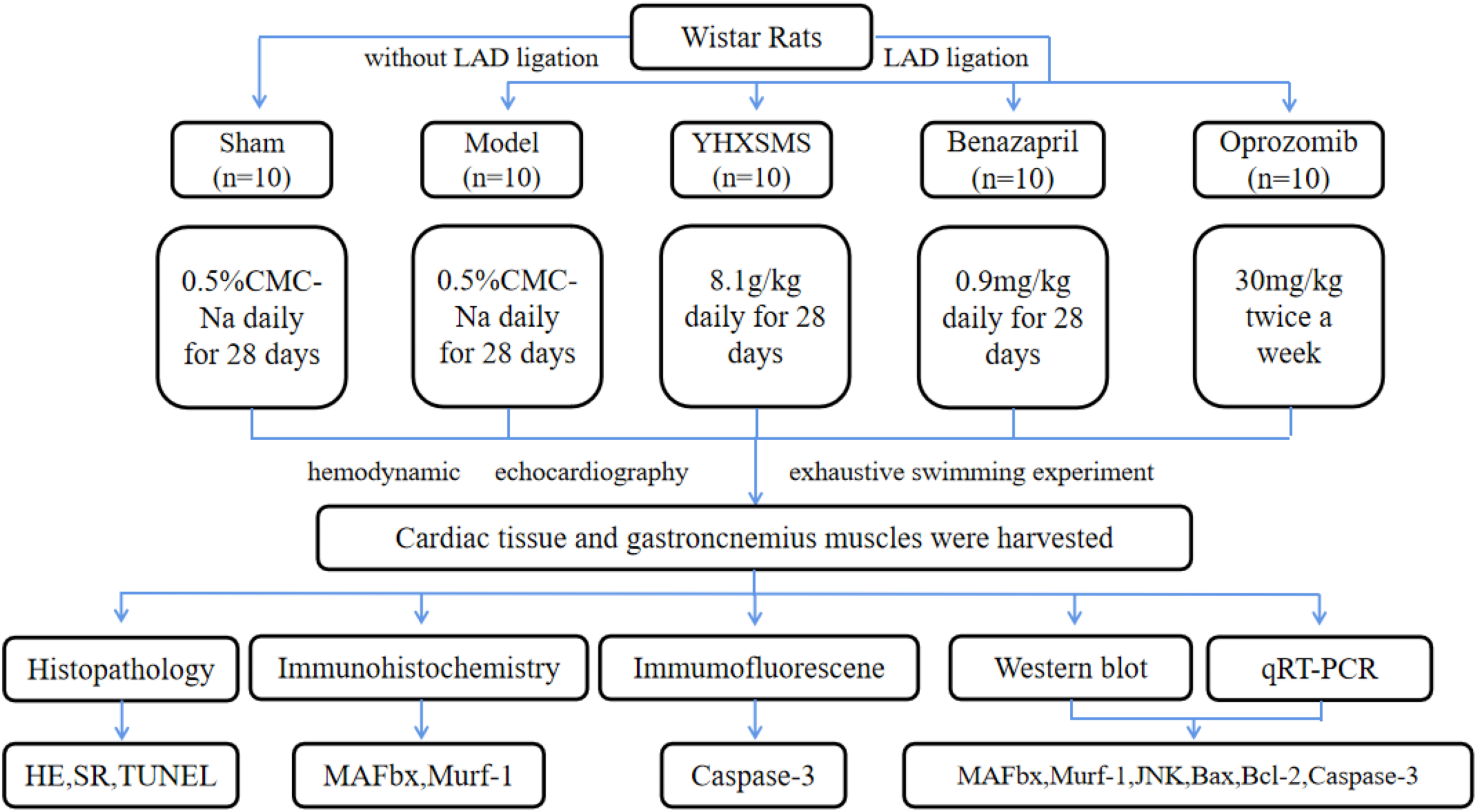
Study designs. Left anterior descending (LAD) coronary artery was ligated to induce the heart failure model. Ten rats in the sham groups underwent identical procedures except that their LAD arteries were not ligated. The rats that were alive after surgery were randomly divided into four groups, with 10 rats in each group.

A total of 100 male Wistar rats (200–240 g) were provided by Beijing Vital River Laboratory Animal Technology Co., Ltd. (Beijing, China); the animal license number was SYXK (Jin) 2019-0002. The rats were given a general diet for adaptation for 7 days. Ten rats that could not swim or had poor swimming ability were eliminated by adaptive swimming training, and the remaining 90 rats were subjected to corresponding experiments (Figure 1). The animal experiment was approved by the Institute of Radiation Medicine Chinese Academy of Medical Sciences (IRM-DWLL-2017046), and all experiments complied with the Guide for the Institutional Animal Care and Use Committee.

### 2.2. Experimental drugs

The optimized new Shengmai powder was composed of *Astmgali radix*, *Codonopsis radix*, *Acanthopanacis Senticosi Radix Et Rhizoma Seu Caulis*, *Trionycis carapax*, *Descurainiae semen*, *Poria*, *Radix ophiopogonis*, *Fructus aurantii*, and *Salviae miltiorrhizae*, which were provided by the First Teaching Hospital of Tianjin University of Traditional Chinese Medicine.

Benazepril was chosen as the control of Western medicine in this study. It belongs to the group of angiotensin-converting enzyme inhibitors, which is the cornerstone and preferred drug for the treatment of heart failure. Benazepril can reduce vascular resistance and improve cardiac output by inhibiting the conversion of angiotensin I to angiotensin II. At the same time, Benazepril can inhibit the activation of the renin–angiotensin–aldosterone system, reduce aldosterone secretion, and reverse ventricular remodeling. Moreover, Benazepril has been confirmed to improve clinical symptoms and increase exercise tolerance in heart failure patients (30). Benazepril (10 mg) was provided by Beijing Novartis Co., Ltd. (Lot No. X2695).

Oprozomib is an effective, orally active proteasome inhibitor that can inhibit 26S proteasome activity and depress the ubiquitin-proteasome signaling pathway. In this study, Oprozomib was chosen as a positive control in this study to verify whether the ubiquitin-proteasome signaling pathway is a common pathway between the cardiac and the skeletal muscle (31). The proteasome inhibitor Oprozomib was provided by MedChemExpress Co., Ltd. (Lot No. Cat.# HY-12113/CS-2767).

### 2.3. Modeling and grouping methods

Ninety healthy rats were anesthetized by administering intraperitoneal injection with Tribromoethanol. The rats were intubated and connected to a ventilator for assisted ventilation. The left anterior descending branch of the coronary artery in the rats was ligated to establish the heart failure models. Ten rats with only threading and no ligation was used as the sham-operated group. All rats were given 100,000 units of penicillin intraperitoneally for 3 days after surgery to prevent infection. The cardiac function of rats was examined at 6 weeks after operation using Vevo 2100 cardiac echocardiography to evaluate the success of modeling [left ventricular ejection fraction (LVEF) ≤ 45%]. According to the LVEF, rats meeting the standard were randomly divided into the following groups: model group, YHXSMS group, Benazepril group, and Oprozomib group, with 10 rats in each group. The sham-operated group and model group were given 4.5 mL/kg of blank solvent (0.5% CMC-Na) by gavage for 4 weeks, once a day. The YHXSMS group was given the optimized new Shengmai powder (8.1 g raw drug/kg) by gavage for 4 weeks, once a day (29, 32). The Benazepril group was given Benazepril (0.9 mg/kg) by gavage for 4 weeks, once a day. The Oprozomib group was given the proteasome inhibitor Oprozomib (30 mg/kg) by gavage twice a week and 4.5 mL/kg blank of solvent by gavage for the remaining days, once a day.

### 2.4. Echocardiography examination

After 4 weeks of administration, the rats were examined for cardiac function using Vevo 2100 cardiac echocardiography. A long-axis measurement package was selected on M-type images to measure the LVEF and left ventricular fraction shortening (FS).

### 2.5. Exhaustive swimming experiment

After 4 weeks of administration, the rats in each group were weighed. Lead strips, with the weight of 5% of the rat’s mass were used, rolled into small pieces, and tied at the root of the rat’s tails. The rats were placed in a plastic sink. The water depth was approximately 3 times the length of the rat’s tail, and the water temperature was (25–29)°C. The timer was immediately started to record the time from when the rats entered the water to the rat’s head sinking into the water for 10 s (i.e., the time of physical exhaustion).

### 2.6. Hemodynamics test

After 4 weeks of administration, four rats in each group were selected with fasting and water deprivation for 12 h, weighed and anesthetized by an intraperitoneal injection of injection of tribromoethanol, fixed on the rat plate, and intubated in the right carotid artery. The catheter was connected to the PowerLab data acquisition and analysis system through a pressure converter to determine the heart rate, left ventricular systolic pressure, left ventricular end-diastolic pressure, left ventricular end-systolic pressure, and maximum rate of increase in the left ventricular pressure (LV + dp/dt max) of rats.

### 2.7. Histopathology

After 4 weeks of administration, the rats were sacrificed. The left ventricular cardiac muscle and gastrocnemius muscle tissue were extracted, and the tissues were fixed with a 4% paraformaldehyde solution. Then, the specimens were made into paraffin-embedded sections with a thickness of 4 μm and the slices were stained with hematoxylin–eosin (HE) and Sirius Red F 3B (SR). The level of fibrosis around the myocardial infarction area and skeletal muscle was observed and the collagen area was measured under a light microscope.

### 2.8. TUNEL detection

The TUNEL method was used to detect apoptosis by following the instructions (G1502-100 T, Servicebio, China). Green fluorescent cell nuclei were regarded as the uniform standard for all positive cells using Image software, while the identically labeled DAPI blue nuclei were regarded as total cells. The number of positive cells in each photograph and the total number of cells in the photograph were calculated, and the percentage of positive cells (positive cell number/total cell number * 100) was calculated as the apoptosis rate (%).

### 2.9. Immunohistochemistry

Immunohistochemical staining was used to assess the protein levels of MAFbx, Murf-1. The paraffin-embedded sections of 4 μm thickness were incubated with 0.3% H_2_O_2_ at indoor temperature, closed with 5% BSA, then incubated with the antibodies of MAFbx and Murf-1 at 4°C overnight, followed by 2 h of incubation at indoor temperature using secondary antibodies. The slices were observed under a light microscope, and three areas of each slice were randomly selected for photography. Image-Pro Plus 6.0 software was applied to analyze the accumulated optical density (IOD) and pixel area (AREA) of each photograph, and the average optical density (AO) was calculated (AO = IOD/AREA). The higher the AO value meant a higher positive expression level.

### 2.10. Immunofluorescence analysis

The activity of Caspase-3 proteasome was measured by the immunofluorescence method. The tissue sections were placed in an ethylene diamine tetraacetic acid (EDTA) antigen repair buffer and repaired in a microwave oven. After washing with PBS, autofluorescence quench was added for 5 min, BSA was incubated at room temperature for 30 min, and then primary antibodies were added at 4°C overnight. Thereafter, DAPI staining solution was added drop by drop, and the slices were sealed and observed under a fluorescence microscope. Nuclei were blue under UV excitation, and the positive expression was a red light labeled with the corresponding fluorescein.

### 2.11. Western blot

The total amount of protein of the rat cardiac muscle tissue and gastrocnemius muscle tissue was extracted, and the protein concentration was determined by the bicinchoninic acid method. The extracted proteins were then separated by way of sodium dodecyl sulfate-polyacrylamide gel electrophoresis (SDS-PAGE), and the proteins were then transferred onto the polyvinylidene fluoride (PVDF) membrane. After blocking with 5% skimmed milk powder for 2 h, primary antibodies MAFbx (1:100, Art. No.sc-166806, Santa, USA), murf-1 (1:1,000, Art. No.bs-2539R, BIOSS, China), JNK (1:1,000, Art. No.PA003084, Cusabio, China), Bcl-2 (1: 1,000, Art. No. ab196495, Abcam, UK), bax (1:5,000, Art. No.ab32503, Abcam, UK), caspase-3 (1:500, Art. No.ab13847, Abcam, UK), GAPDH (1:2,500, Art. No.ab9485, Abcam, UK) were added separately and incubated at 4°C overnight. The next day, after 2 h incubation with secondary antibodies at indoor temperature, the target bands were exposed to an ultrasensitive multifunction imager, and then the expression intensity of each target protein/GAPDH was analyzed using ImageJ software 1.8.0.

### 2.12. Quantitative real-time PCR

Total RNA was extracted from cardiac and gastrocnemius tissues using the HP Total RNA Kit (R6812-01, Omega Bio-tek, Norcross, GA), and the RNA was reversed to cDNA using HiScript® Q RT SuperMix (R122-01, Vazyme, China), followed by quantitative real-time PCR (qRT-PCR) using SYBR qPCR Master Mix (Q711-02, Vazyme, China). The primer sequences used in this study are given in Table 1. The relative quantitative expression of mRNA 2^−△△CT^ was analyzed with the circulation threshold.

**Table 1:** The sequence of forward and reverse primers.

	Forward (5ʹ-3ʹ)	Reverse (3ʹ-5ʹ)
GAPDH	CCCCTACATTTGGAGCCTGG	TTGCGACCCACGTAGTAGAC
MAFbx	GGTCCAGAGAGTCGGCAAGT	GGAGCAGCTCTCTGGGTTGT
Murf-1	TCCAAGGACAGAAGACTGAACT	TGGAAGCTTCTACAATGCTCTT
JNK	AAGACAGGGGAGCAGACG	GGTGGCAAGAAGGTGGAG
Caspase-3	ACGAACGGACCTGTGGACCTG	GTTTCGGCTTTCCAGTCAGACTCC
Bax	GACGCATCCACCAAGAAGCTGAG	GCTGCCACACGGAAGAAGACC
Bcl-2	TGGAGAGCGTCAACAGGGAGATG	GGTGTGCAGATGCCGGTTCAG

## 3. Results

### 3.1. Effects of the optimized new Shengmai powder on cardiac function and exercise tolerance in rats

**Figure 2:**
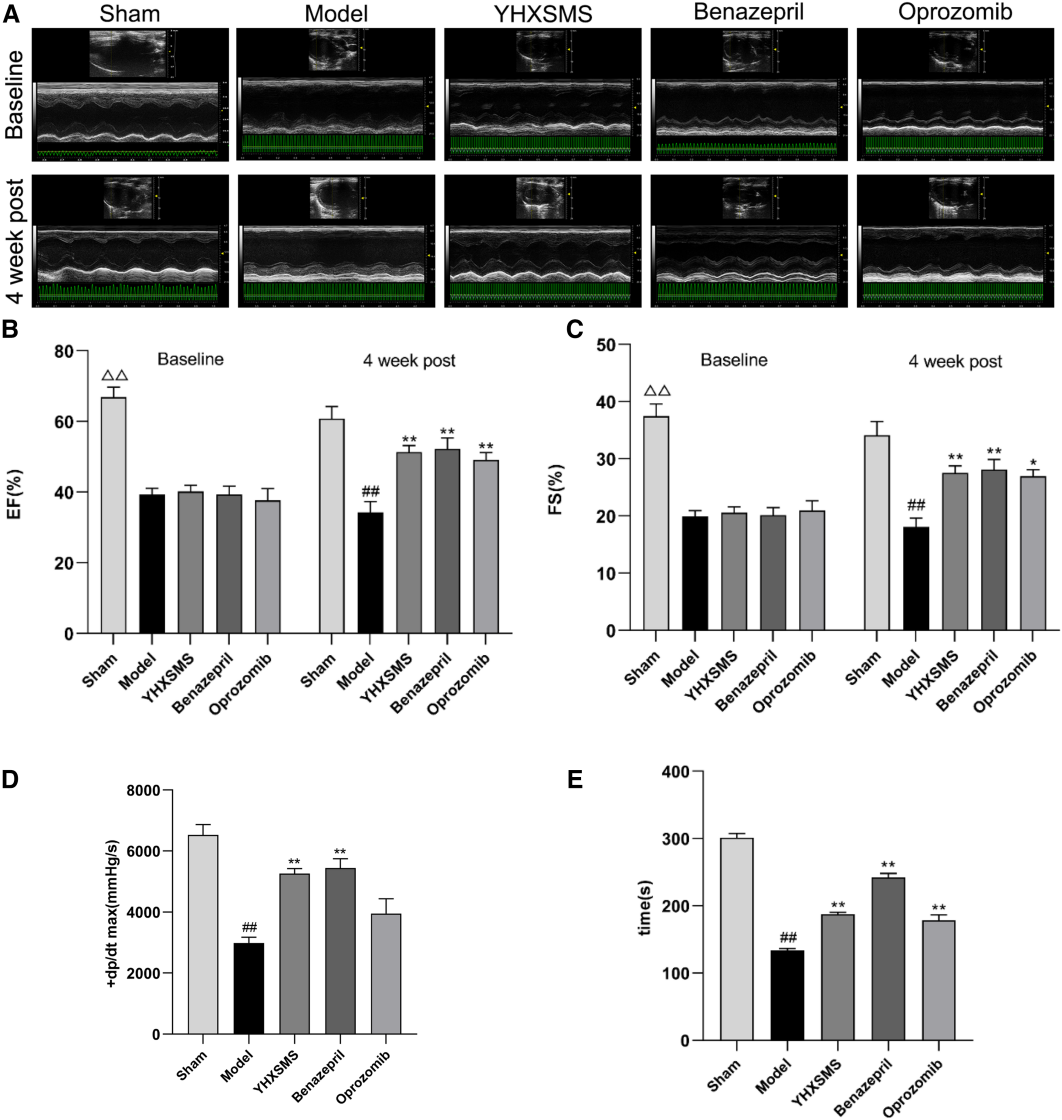
Changes in cardiac function and exercise tolerance of rats in each group before intragastric administration (i.e., baseline) and after intragastric administration (i.e., 4 week post). (**A**) Echocardiogram of rats in each group before and after intragastric administration. (**B**) Changes in the LVEF of rats in each group before and after intragastric administration. (**C**) Changes in the FS of rats in each group before and after intragastric administration. (**D**) Hemodynamic changes in rats in each group after intragastric administration. (**E**) Changes in the swimming time of exhaustion of rats in each group after intragastric administration. At baseline, the sham-operated group compared with the rest of the rats, ^△△^*P* < 0.01. 4 week post, compared with the sham-operated group, ^##^*P* < 0.01, compared with the model group, *P < 0.05, **P < 0.01.

Before administration, compared with the sham-operated group, the levels of the LVEF and FS of rats in the other groups decreased, and the differences were statistically significant (*P* < 0.01). Meanwhile, there was no statistical difference among the model group, YHXSMS group, Benazepril group, and Oprozomib group in terms of the levels of the LVEF and FS before administration (*P* > 0.05). After 4 weeks of administration, these levels increased in the YHXSMS, Benazepril, and Oprozomib groups compared with the model group (*P* < 0.05, *P* < 0.01). Hemodynamics showed that the levels of LV + dp/dt max (mmHg/s) and left heart function decreased in the model group compared with the sham-operated group (*P* < 0.01), while the level of LV + dp/dt max (mmHg/s) in the YHXSMS group and Benazepril group increased compared with the model group (*P* < 0.01). As for exercise tolerance, there was a shortened swimming time in the model group compared with the sham-operated group (*P* < 0.01), while it was an extended one in the YHXSMS, Benazepril, and Oprozomib groups compared with that in the model group (*P* < 0.01) (Figure 2).

### 3.2. Effects of the optimized new Shengmai powder on the structure of the cardiac muscle and skeletal muscle in rats

HE staining showed that the myocardial fibers were arranged densely and neatly, the nuclei were clearly visible, the cytoplasm was uniformly red-stained, and the cell membrane was intact with clear boundaries in the sham-operated group. Myocardial fibers in the marginal area of myocardial infarction were disorganized with partial rupture, and there was a significant widening of the myocardial gap with varying shades of cytoplasmic staining in the model group. In the YHXSMS, Benazepril, and Oprozomib groups, the myocardial fibers were relatively regular in shape, with an inconspicuous widening of the muscle gap and a relatively uniform cytoplasmic staining. The skeletal muscle cells in the sham-operated group had normal outlines, complete cytosolic membranes, neatly arranged myogenic fibers, and clear structures. In contrast, the skeletal muscle fibers in the model group were disorganized and the muscle fiber gaps were broad, accompanied by a disruption in integrity and continuity. There was a significant reduction in pathological changes in the skeletal muscle in the YHXSMS, Benazepril, and Oprozomib groups compared with the model group.

**Figure 3:**
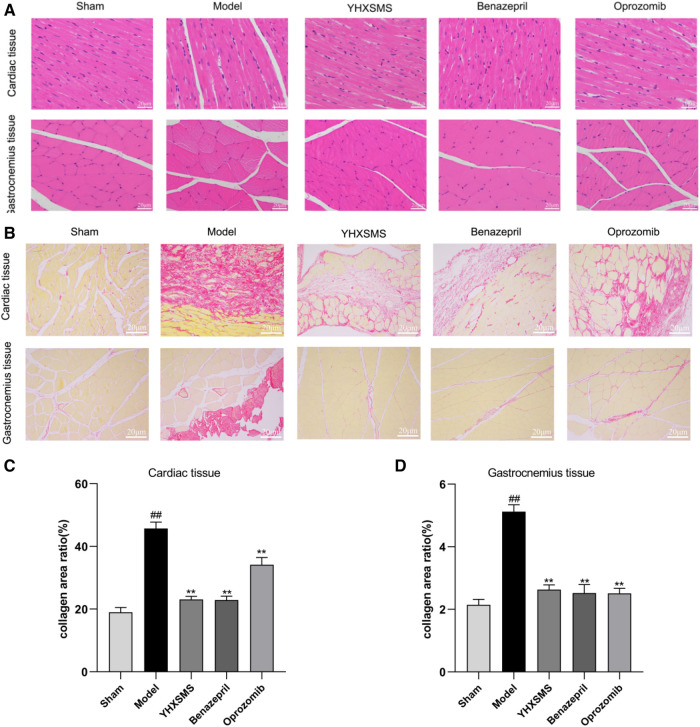
HE and SR staining of the myocardium and skeletal muscle of the rats. (**A**) HE staining of the rats’ myocardium and skeletal muscle. (**B**) SR staining of the rats’ myocardium and skeletal muscle. (**C**) Changes in the collagen area of the myocardial tissue of rats in each group. (**D**) Changes in the collagen area of the skeletal muscle of rats in each group. Compared with the sham-operated group, ^##^*P* < 0.01. Compared with the model group, **P < 0.01.

SR staining showed that there were few red collagen fibers in the sham-operated group, and the red collagen area of the myocardial tissue increased in the model group compared with the sham-operated group (*P* < 0.01). Compared with the model group, there was a reduction in the red collagen area of the myocardial tissue in the YHXSMS, Benazepril, and Oprozomib groups (*P* < 0.01). Skeletal muscle tissue collagen fibers were rare in the sham-operated group, while there was a proliferation of collagen fibers and a reduction in the cross-sectional area of the skeletal muscle in the model group (*P* < 0.01). Compared with the model group, there was a reduction in the red collagen area of the skeletal muscle tissue in the YHXSMS, Benazepril, and Oprozomib groups (*P* < 0.01) (Figure 3).

### 3.3. Effects of the optimized new Shengmai powder on TUNEL staining and the apoptosis rate of cardiac and skeletal muscle cells in rats

**Figure 4:**
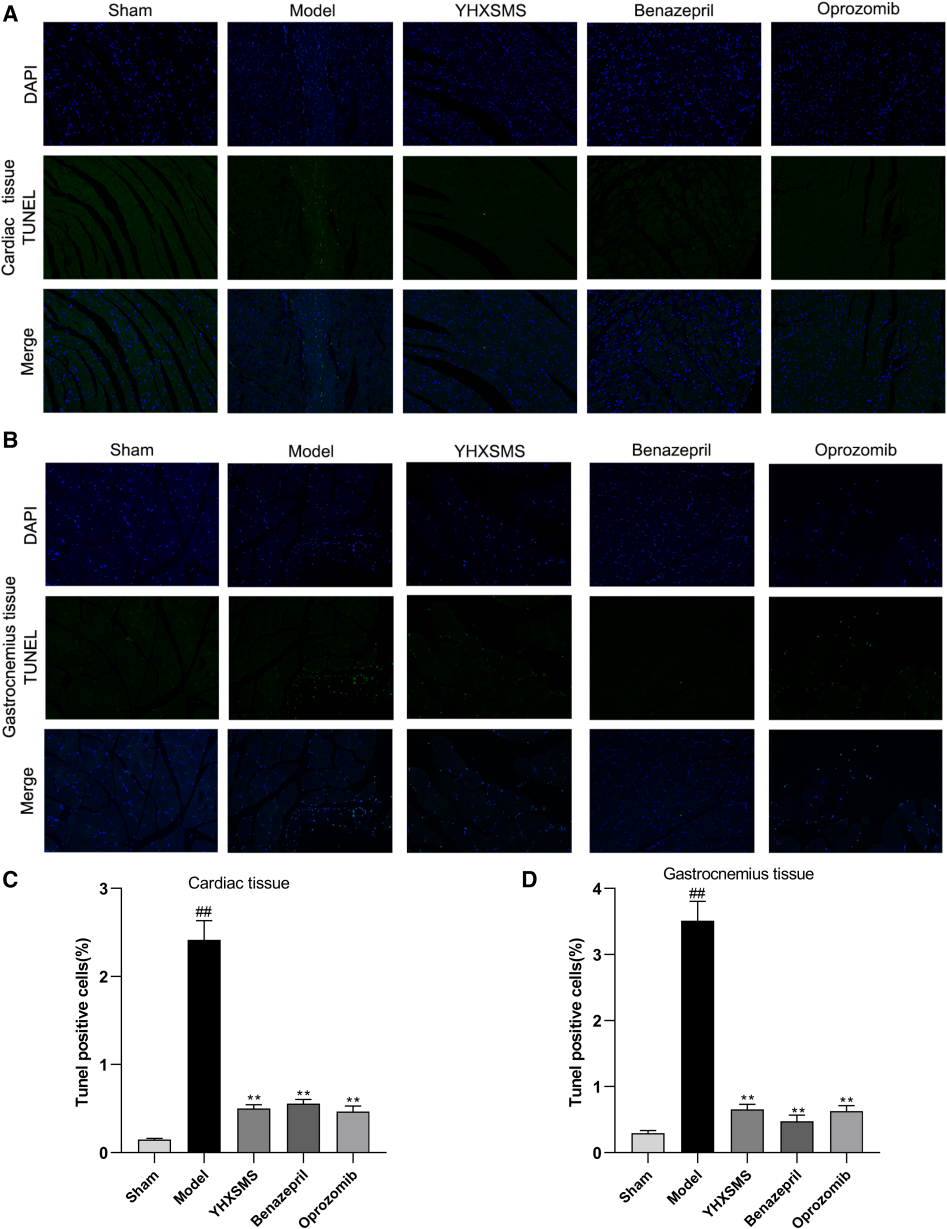
Comparison of TUNEL staining and the apoptosis rate between the cardiac and the skeletal muscle cells of rats. (**A**) TUNEL staining of the cardiac muscle cells of rats. (**B**) TUNEL staining of the skeletal muscle cells of rats. (**C**) Comparison of apoptosis in the cardiac muscle cells of rats. (**D**) Comparison of apoptosis in the skeletal muscle cells of rats. Compared with the sham-operated group, ^##^*P* < 0.01. Compared with the model group, **P < 0.01.

TUNEL staining showed that a small number of green fluorescent cells were visible in the sham-operated group, while a large number of positive green fluorescent cells were visible in the model group with an increased apoptosis rate (*P* < 0.01). Compared with the model group, the YHXSMS, Benazepril, and Oprozomib groups had fewer positive green fluorescent cells and a lower apoptosis rate (*P* < 0.01) (Figure 4).

### 3.4. Effects of the optimized new Shengmai powder on the expression of MAFbx and Murf-1 protein of the cardiac and skeletal muscles in rats by immunohistochemistry

**Figure 5:**
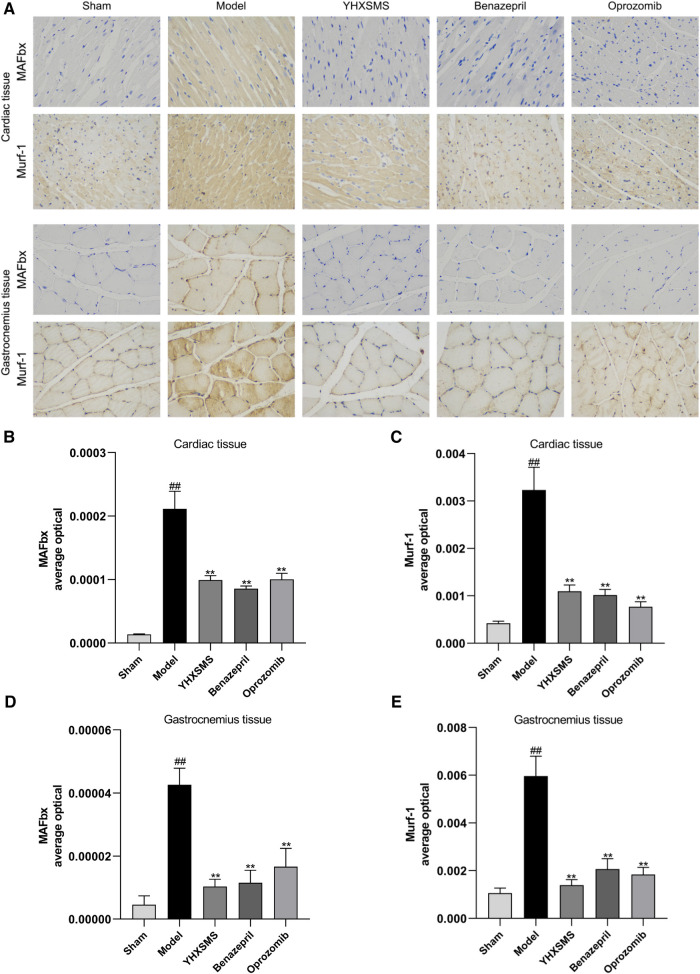
Immunohistochemical results of the expression of MAFbx and murf-1 proteins in the myocardium and skeletal muscle of rats. (**A**) Staining of MAFbx and Murf-1 in the myocardium and skeletal muscle. (**B**) Changes in MAFbx protein expression in the myocardium. (**C**) Changes in Murf-1 protein expression in the myocardium. (**D**) Changes in MAFbx protein expression in the skeletal muscle. (**E**) Changes in Murf-1 protein expression in the skeletal muscle. Compared with the sham-operated group, ^##^*P* < 0.01. Compared with model group, **P < 0.01.

Immunohistochemistry showed that there were increased MAFbx and Murf-1 protein expressions in the model group compared with those in the sham-operated group (*P* < 0.01). Compared with the model group, there were reduced MAFbx and Murf-1 protein expressions in the YHXSMS, Benazepril, and Oprozomib groups (*P* < 0.01) (Figure 5).

### 3.5. Effects of the optimized new Shengmai powder on apoptosis gene Caspase-3 of cardiac and skeletal muscles in rats

**Figure 6:**
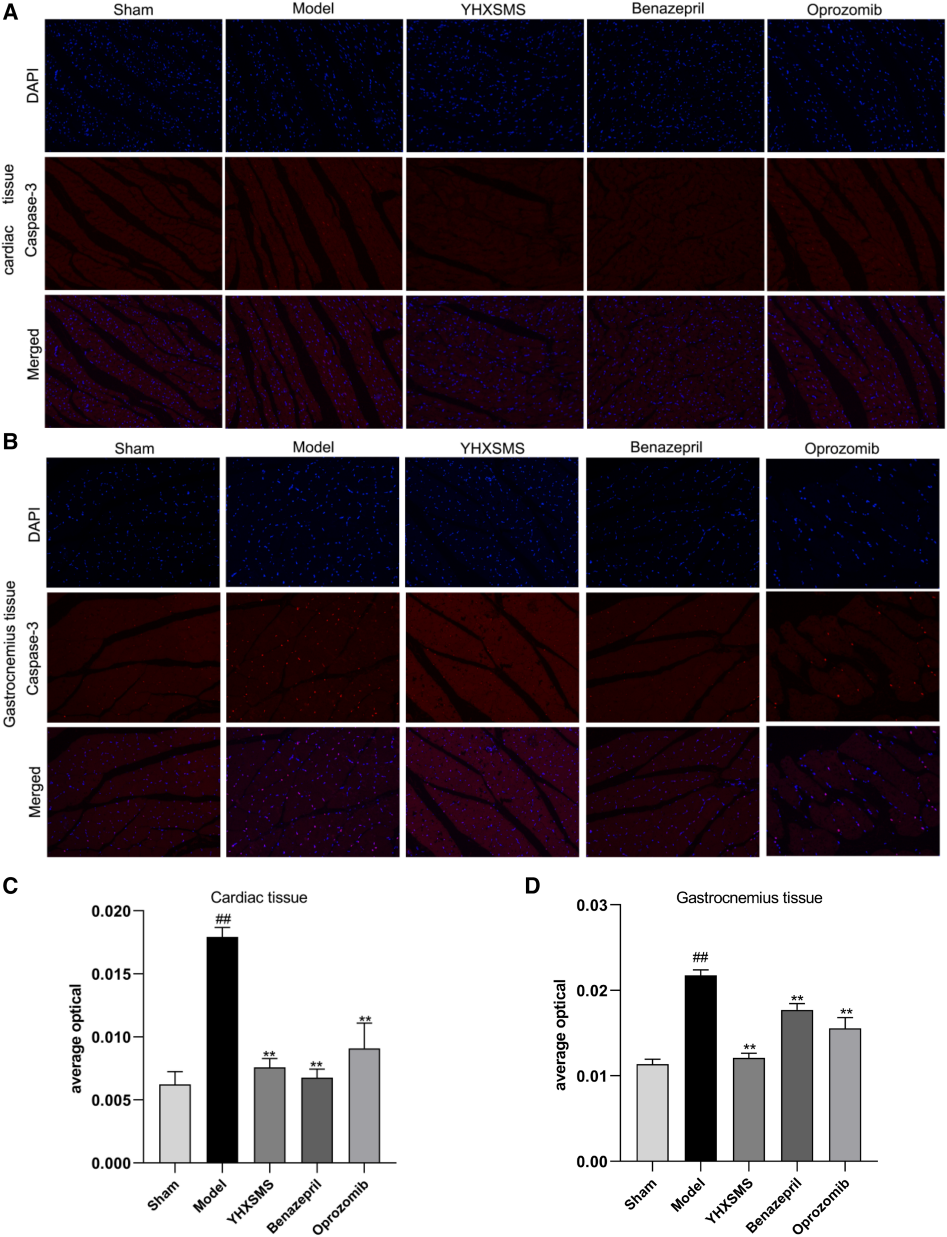
Results of immunofluorescence analysis of caspase-3. (**A**) Fluorescent staining of caspase-3 in cardiac muscle cells. (**B**) Fluorescent staining of caspase-3 in skeletal muscle cells. (**C**) Comparison of Caspase-3 activity in cardiac muscle cells. (**D**) Comparison of Caspase-3 activity in skeletal muscle cells. Compared with the sham-operated group, ^##^*P* < 0.01. Compared with the model group, *P < 0.05, **P < 0.01.

The results of immunofluorescence analysis showed that more nuclei were stained with red fluorescence in the cardiac and skeletal muscles of rats in the model group than in the sham-operated group (*P* < 0.01). Compared with the model group, there was a lower number of nuclei with red fluorescence in the YHXSMS, Benazepril, and Oprozomib groups (*P* < 0.05, *P* < 0.01) (Figure 6).

### 3.6. Effect of the optimized new Shengmai powder on the expression of UPS pathway-related proteins in the cardiac and skeletal muscles of rats

**Figure 7:**
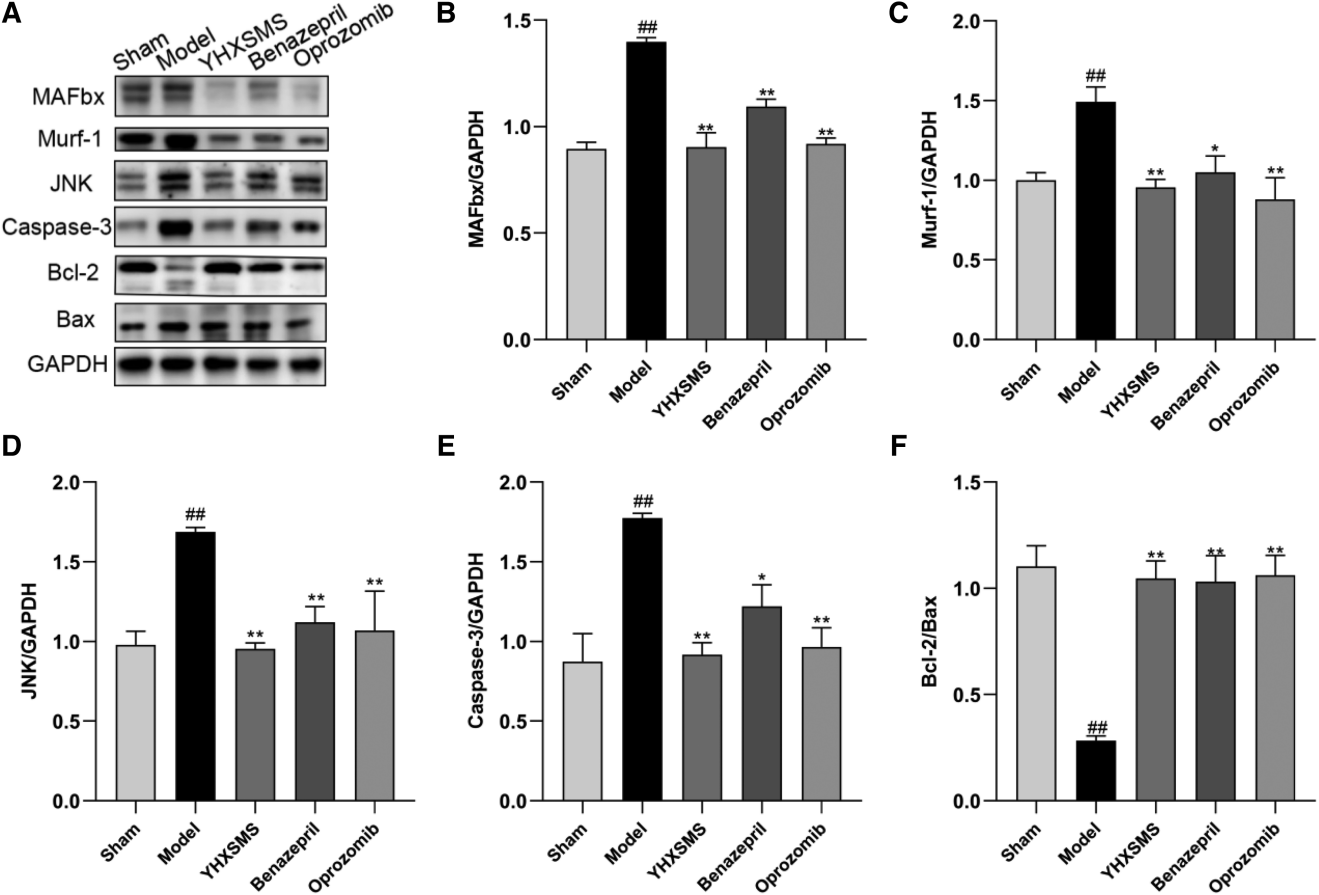
Expression of cardiac muscle–related proteins. (**A**) The results of Western blot analysis. (**B–F**) Expression levels of MAFbx, Murf-1, JNK, Caspase-3, and Bcl-2/Bax in each group. Compared with the sham-operated group, ^##^*P* < 0.01. Compared with the model group, *P < 0.05, **P < 0.01.

**Figure 8:**
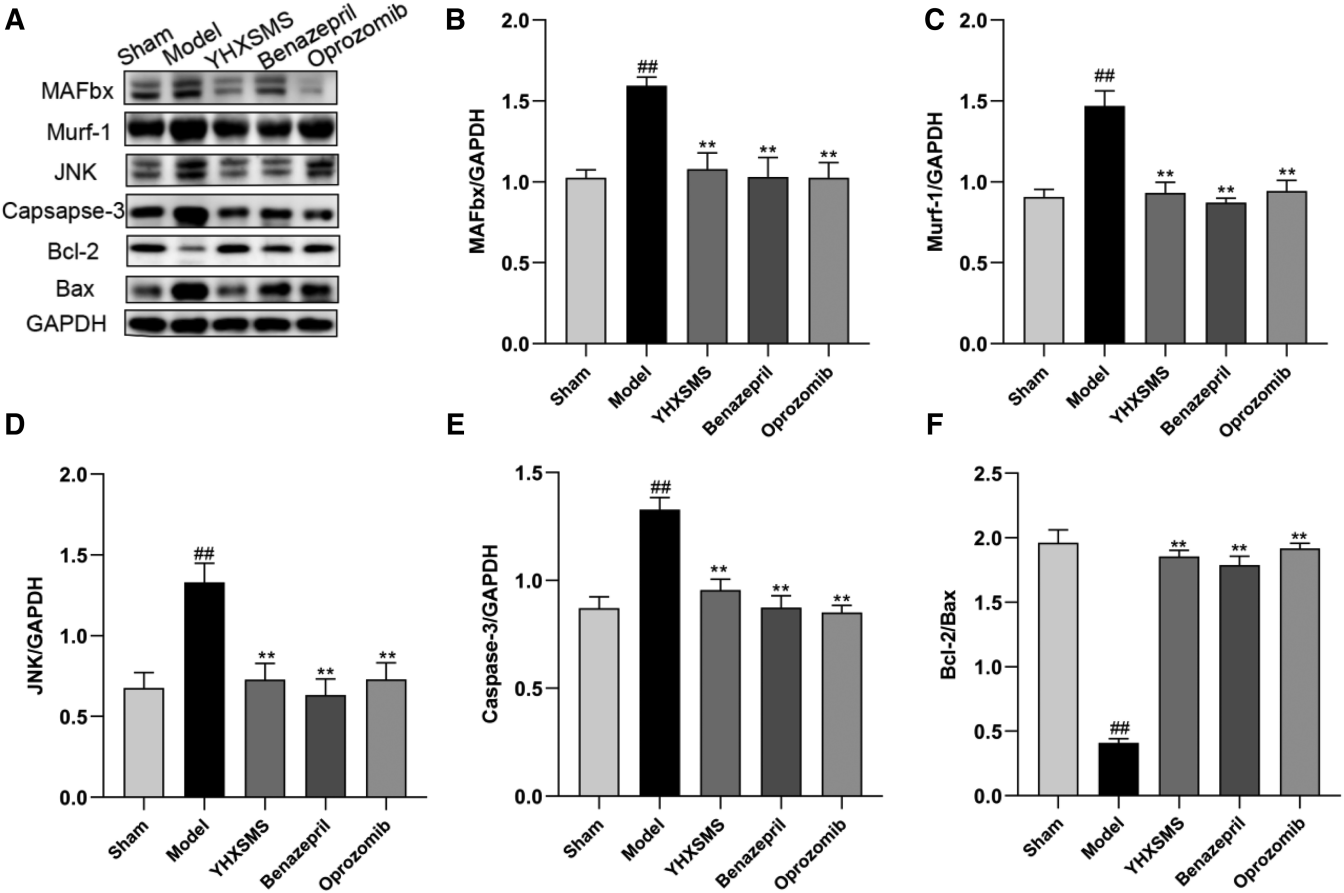
Expression of skeletal muscle–related proteins. (**A**) The results of Western blot analysis. (**B–F**) Expression levels of MAFbx, Murf-1, JNK, Caspase-3, and Bcl-2/Bax in each group. Compared with the sham-operated group, ^##^*P* < 0.01. Compared with the model group, *P < 0.05, **P < 0.01.

**Figure 9:**
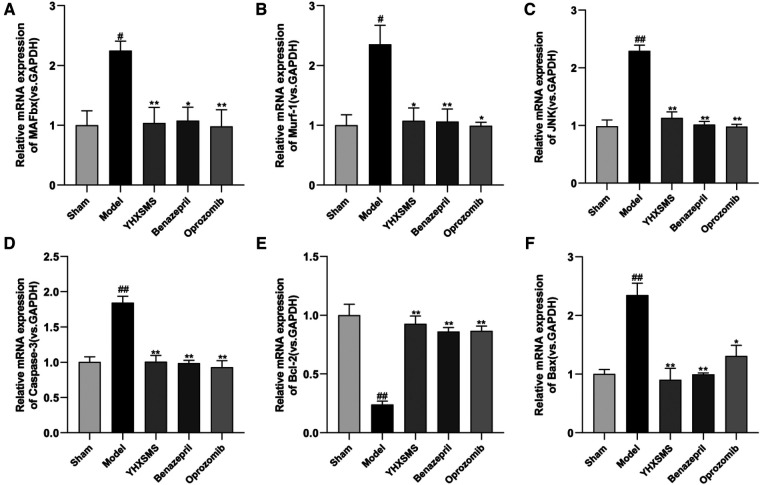
Expression of myocardial-related mRNA. (**A–F**) Expression levels of MAFbx, Murf-1, JNK, Caspase-3, Bcl-2, and Bax in each group. Compared with the sham-operated group, ^#^*P* < 0.05, ^##^*P* < 0.01. Compared with the model group, *P < 0.05, **P < 0.01.

**Figure 10:**
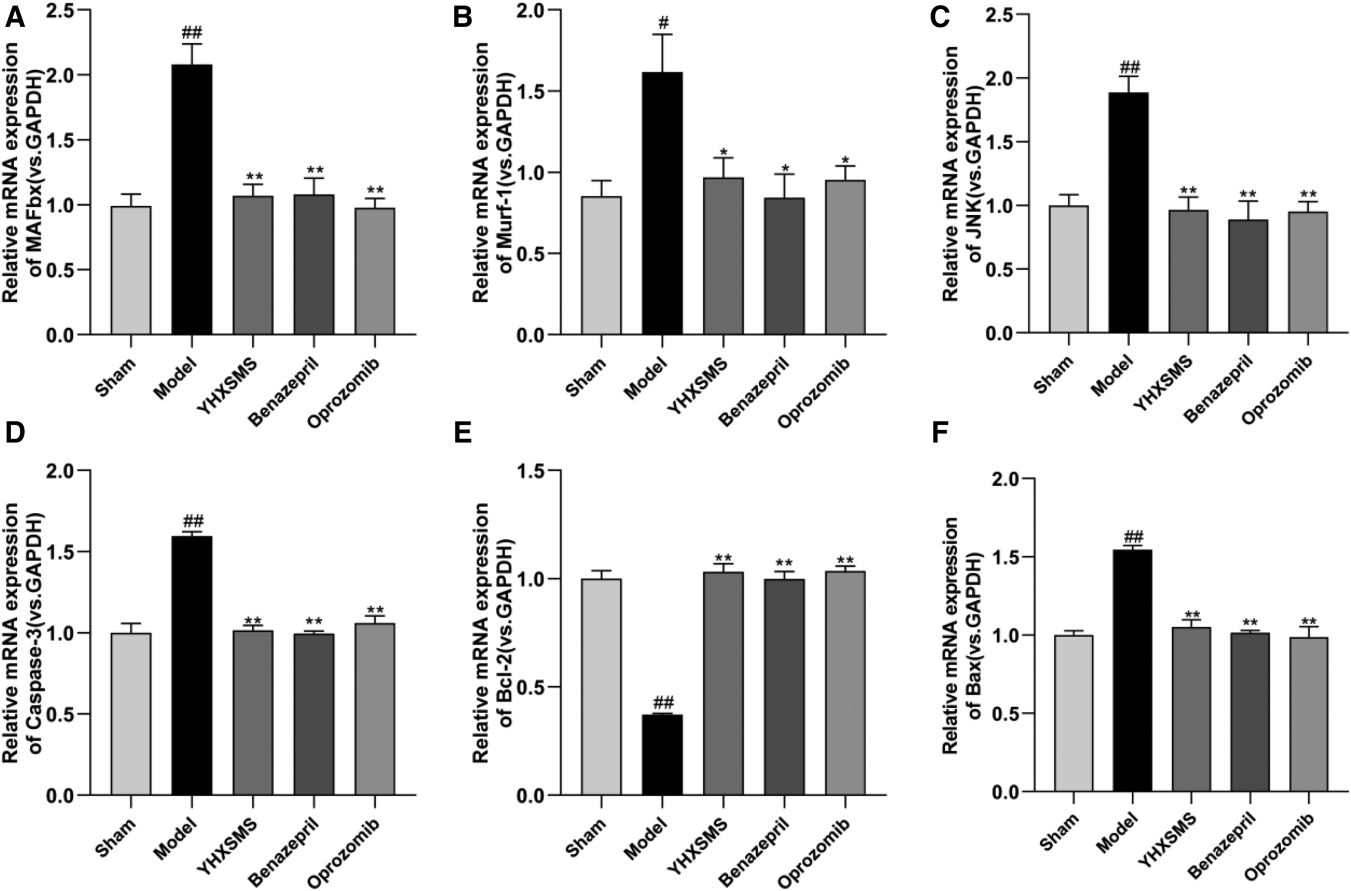
Expression of skeletal muscle–related mRNA. (**A–F**) Expression levels of MAFbx, Murf-1, JNK, Caspase-3, Bcl-2, and Bax in each group. Compared with the sham-operated group, ^#^*P* < 0.05, ^##^*P* < 0.01. Compared with the model group, *P < 0.05, **P < 0.01.

The results of Western blot analysis showed that there were increased levels of MAFbx, Murf-1, JNK, and Caspase-3 and a decreased expression of Bcl-2/Bax in the model group compared with those in the sham-operated group (*P* < 0.01). In contrast to the model group, there were decreased MAFbx, Murf-1, JNK, and Caspase-3 levels and an increased Bcl-2/Bax expression in the YHXSMS, Benazepril, and Oprozomib groups (*P* < 0.05, *P* < 0.01) (Figures 7, 8). The results of qRT-PCR showed that there were increased MAFbx, Murf-1, JNK, Caspase-3, and Bax levels and a decreased Bcl-2 expression in the model group compared with those in the sham-operated group (*P* < 0.05, *P* < 0.01). In contrast to the model group, there were decreased MAFbx, Murf-1, JNK, Caspase-3, and Bax levels and an increased Bcl-2 expression in the YHXSMS, Benazepril, and Oprozomib groups (*P* < 0.05, *P* < 0.01) (Figures 9, 10).

## 4. Discussion

The UPS consists of ubiquitin, ligases, and 26S proteasome, which has a wide range of biological functions to regulate protein degradation and apoptosis (33, 34). The formation of ubiquitin–protein complexes requires three enzymes that can catalyze a continuous reaction of ubiquitin, including the E1 ubiquitin-activating enzyme, E2 ubiquitin-conjugating enzyme, and E3 ubiquitin ligase. In particular, E3 ubiquitin ligase can specifically recognize and bind target proteins and catalyze the degradation of proteins by the 26S proteasome. Studies have found that E3 ubiquitin ligase, MAFbx, and Murf-1 are specifically expressed in the myocardium and skeletal muscle, which are involved in ventricular remodeling and skeletal muscle atrophy and other pathological processes.

**Figure 11:**
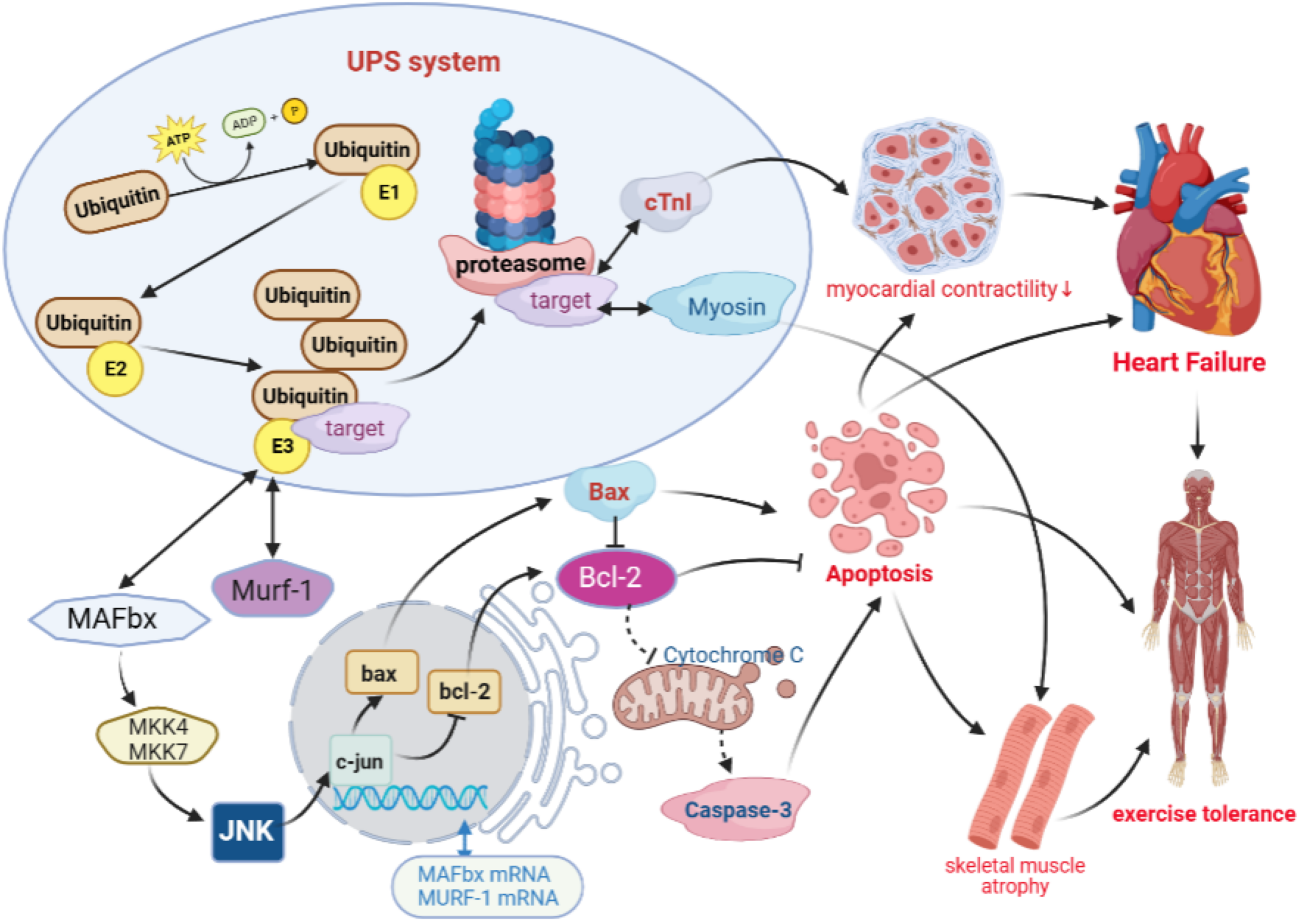
Ubiquitin-proteasome signaling pathway.

It has been shown that a high expression of MAFbx in cardiomyocytes enhances the phosphorylation of JNK. Furthermore, being the regulator upstream of Bcl-2 and Bax, JNK can decrease the expression of Bcl-2, increase the expression of Bax, and increase the activity of caspases-3, which are involved in apoptosis (35, 36). On the other hand, Murf-1 in the myocardial tissue can increase cTnI degradation through ubiquitination, which causes a reduction in myocardial contractility and leads to ventricular remodeling (19). Meanwhile, the results of animal experiments have confirmed that MAFbx is involved in apoptosis and fast and slow muscle fiber transitions through the regulation of JNK signaling in the skeletal muscle (37, 38). Murf-1 is involved in the shear degradation of Myosin, which leads to a decrease in muscle fiber contractility and exercise tolerance (39, 40). However, previous studies on decreased exercise tolerance in heart failure mostly focused on single isolated studies to improve ventricular remodeling and increase myocardial contractility, without linking non-cardiogenic skeletal muscle lesions as an organic whole. These studies were based on the hypothesis that “UPS is a common pathway of cardiac muscle and skeletal muscle degradation and apoptosis in heart failure, and the optimized new Shengmai powder can effectively improve ventricular remodeling and skeletal frontal myopathy by regulating both myocardial and skeletal muscle targets simultaneously through the UPS signaling pathway, thus improving exercise tolerance.” The results showed that there was a reduction in cardiac function, a decrease in exercise tolerance, damage to cardiac and skeletal muscle fibers, a proliferation in collagen tissue, and an increased apoptosis in rats with heart failure. A further mechanism study revealed that there was an overactivation of the UPS pathway in the rat cardiac and skeletal muscle tissues, with increased expressions of MAFbx and Murf-1, which, in turn, activated the downstream JNK signaling pathway responsible for apoptosis and differentiation (41–44), triggering the downregulation of bcl-2 and upregulation of bax, and activating caspase-3 to promote apoptosis (Figure 11). However, the optimized new Shengmai powder could exert antiapoptotic effects on the myocardial and skeletal muscle cells and improve myocardial contractility and exercise tolerance by inhibiting the overactivation of the UPS pathway, downregulating MAFbx and Murf-1 overexpression, decreasing the activation of the JNK signaling pathway, increasing bcl-2 expression, and inhibiting bax and caspase-3 levels.

In response to the multifactorial, multilinked, and multisite characteristics of the decline in exercise tolerance in heart failure (45), the optimized new Shengmai powder could modulate cardiac and skeletal muscles together through the UPS pathway. Modern pharmacological studies have shown that the *Astragalus membranaceus* in this powder can promote cardiac microvascular formation, ameliorate cardiomyocyte hypertrophy, alleviate excessive oxidative stress, modify energy metabolism disorders, and reduce cardiomyocyte apoptosis (46, 47). Its main component, astragaloside IV, can ameliorate the pathological changes and fibrotic deposition of myocardial infarction and improve cardiomyocyte survivability (48). *Radix Codonopsis pilosulae* can inhibit platelet aggregation, enhance microcirculation, and regulate blood lipids (49), which can effectively improve myocardial energy metabolism, reduce inflammatory response, and protect the myocardium (50). *Acanthopanax senticosus* can resist myocardial ischemia, arrhythmia, and oxidation reaction (51). *Acanthopanax senticosus saponins* can inhibit apoptosis by downregulating Caspase-3 and Bax protein expressions (52). *Salvia miltiorrhiza* is known for its wide range of benefits such as preventing cardiovascular diseases, reducing myocardial damage, as well as fighting inflammation and antioxidants (53, 54). *Semen lepidii* can ameliorate cellular oxidative stress, inhibit endogenous apoptotic pathways, and effectively protect the damaged H9C2 cardiomyocytes (55). *Ophiopogon japonicus* is able to resist the formation of thrombosis, decrease blood sugar, and regulate immunity (56), while its active ingredient, methylophiopogonanone A, can reduce the myocardial infarct area and inhibit cardiomyocyte apoptosis (57). *Fructus aurantii* has the ability to inhibit the myocardial remodeling and inflammatory response of vascular endothelial cells and also inhibit platelet aggregation (58). The optimized new Shengmai powder contains various drug components, which is, therefore, worthy of further study.

## 5. Conclusion

This study showed that the optimized new Shengmai powder could exert antiapoptotic effects on myocardial and skeletal muscle cells and improve myocardial contractility and exercise tolerance by inhibiting the overactivation of the UPS pathway, downregulating the overexpression of MAFbx, and Murf-1, inhibiting the activation of the JNK signaling pathway, upregulating bcl-2 expression, and decreasing bax and caspase-3 levels. However, it is necessary to further screen the specific compound components of the optimized new Shengmai powder and explore how they produce this protective function.

## Data Availability

The raw data supporting the conclusions of this article will be made available by the authors without undue reservation.
